# Comparison of Genetic and Epigenetic Alterations of Primary Tumors and Matched Plasma Samples in Patients with Colorectal Cancer

**DOI:** 10.1371/journal.pone.0126417

**Published:** 2015-05-06

**Authors:** Elisa Danese, Anna Maria Minicozzi, Marco Benati, Martina Montagnana, Elisa Paviati, Gian Luca Salvagno, Gabriel Lima-Oliveira, Milena Gusella, Felice Pasini, Giuseppe Lippi, Gian Cesare Guidi

**Affiliations:** 1 Laboratory of Clinical Biochemistry, Department of Life and Reproduction Sciences, University Hospital of Verona, Verona, Italy; 2 National Centre for Bowel Research and Surgical Innovation (NCBRSI), Academic Surgical Unit, Barts and The London NHS Trust, Queen Mary University of London, London, United Kingdom; 3 Oncology Department, Laboratory of Pharmacology and Molecular Biology, Rovigo General Hospital, Trecenta, Rovigo, Italy; 4 Department of Medical Oncology, Rovigo Hospital, Rovigo, Italy; 5 Laboratory of Clinical Chemistry and Hematology, Academic Hospital of Parma, Parma, Italy; Queen Mary Hospital, HONG KONG

## Abstract

**Background:**

Although recent advances in circulating DNA analysis allow the prediction of tumor genomes by noninvasive means, some challenges remain, which limit the widespread introduction of cfDNA in cancer diagnostics. We analyzed the status of the two best characterized colorectal cancer (CRC) genetic and epigenetic alterations in a cohort of CRC patients, and then compared the degree to which the two patterns move from tissue to plasma in order to improve our understanding of biology modulating the concordance between tissues and plasma methylation and mutation profiles.

**Methods:**

Plasma and tumor tissues were collected from 85 patients (69±14 years, 56 males). *KRAS* and *SEPT9* status was assessed by allele refractory mutation system quantitative PCR and quantitative methylation-specific PCR, respectively. Six of the most common point mutations at codon 12 and 13 were investigated for *KRAS* analysis.

**Results:**

*KRAS* mutations and *SEPT9* promoter methylation were present in 34% (29/85) and in 82% (70/85) of primary tumor tissue samples. Both genetic and epigenetic analyses of cfDNA revealed a high overall concordance and specificity compared with tumor-tissue analyses. Patients presenting with both genetic and epigenetic alterations in tissue specimens (31.8%, 27/85) were considered for further analyses. The median methylation rates in tumour tissues and plasma samples were 64.5% (12.2–99.8%) and 14.5% (0–45.5%), respectively. The median *KRAS* mutation load (for matched mutations) was 33.6% (1.8–86.3%) in tissues and 2.9% (0–17.3) in plasma samples. The plasma/tissue (p/t) ratio of *SEPT9* methylation rate was significantly higher than the p/t ratio of *KRAS* mutation load, especially in early stage cancers (p=0.0108).

**Conclusion:**

The results of this study show a discrepant rate of epigenetic vs. genetic alterations moving from tissue to plasma. Many factors could affect mutation cfDNA analysis, including both presence of tumor clonal heterogeneity and strict compartmentalization of *KRAS* mutation profile. The present study highlights the importance of considering the nature of the alteration when analyzing tumor-derived cfDNA.

## Introduction

Evidence that tumor specific genetic and epigenetic alterations can be detected in circulating DNA extracted from plasma of cancer patients has shown promise for improving early diagnosis, prognostication and disease monitoring. The overarching goal of utilizing cell free DNA as a biomarker entails medical practice optimization, personalized medicine development, and quality of life improvement due to the minimal invasiveness of blood testing. However, the authentication of actual clinical validity of various cell-free DNA (cfDNA) alterations as putative cancer biomarkers in clinical practice remains challenging [1]. The leading issue is currently represented by the fact that circulating DNA fragments carrying tumor specific alterations represent a variable and generally small fraction of the total circulating DNA, thus generating a high variability in the concordance rate between alteration patterns detectable in tissue of primary tumors and corresponding plasma.

The factors influencing the quantitative as well as the qualitative changes of cfDNA with respect to tissues of cancer patients are many and not yet fully explored so far. However, efforts during the last decade have led to important advances.

By evaluating the methylation pattern of the *PCDH10* gene in tissue and plasma of patients with colorectal cancer (CRC) we have recently demonstrated that the methylation rate detected in plasma increased with enhanced methylation rate in tumour tissues only in early-stage cancers, whereas this correlation was apparently lost in advanced cancers. Moreover, we showed that the degree of cfDNA methylation was associated with some characteristics of cfDNA, such as its concentration and integrity, and that these correlations varied in strength and direction in parallel with the tumour stage [2].

In the last two years two independent research groups showed that the possibility to detect tumor specific cfDNA in plasma of CRC patients largely depends on the sensitivity of the PCR-based method for short mutated sequences [3–5], thus emphasizing the importance of minimizing the assay length when analyzing highly fragmented cfDNA, such as in the setting of cancer patients.

Intratumoral heterogeneity and clonal evolution during progression are further issues complicating the use of cfDNA as liquid biopsy for cancer, since both factors generate remarkable differences in the proportion and pattern of aberrations detectable in primary tumor and circulating DNA [6,7].

According to this evidence, different technical and biological aspects should be considered when analysing the variable concordance between tissue and plasma alterations in cancer patients, not least the nature of the underlying alterations.

Both epigenetic and genetic alterations are well-known aberrations involved in colorectal carcinogenesis. Given their enormous potential as biomarkers in CRC diagnosis, staging, prognosis and response to treatment, they have been extensively investigated in the last decade. However, a critical comparison of their status in tissue and cfDNA is lacking. Therefore, this study was aimed to analyze the status of the two best characterized genetic and epigenetic alterations of CRC (i.e., *KRAS* mutation and *SEPT9* promoter methylation) in a cohort of CRC patients, in order to improve our understanding of the biological aspects modulating the concordance between tissues and plasma methylation and mutation profiles. Then, we also compared the degree to which the genetic and the epigenetic patterns move from tissue to plasma.

## Material and Methods

### Patients and Samples

The study cohort included 85 consecutive patients undergoing surgery for CRC at the University Hospital of Verona (Italy) between January 2010 and December 2010. Blood specimens were collected before surgical resection. Tumor samples were obtained during surgery, immediately frozen in liquid nitrogen and stored at -80°C. Histological diagnosis and tumor stage were assessed according to the 2000 World Health Organization (WHO) classification system for tumors of digestive system and the American Joint Committee on Cancer (AJCC) staging system, respectively [8]. Only patients with primary colorectal adenocarcinomas untreated with neoadjuvant radio-chemotherapy were included in the study. All subjects gave a written consent for being enrolled in this investigation. The study was approved by the local ethical committee (Department of Life and Reproduction Sciences, University of Verona) and performed in accord with the Helsinki Declaration of 1975. Clinical information was obtained from medical records.

### DNA isolation from plasma and tissue samples

Blood samples were collected in 7 mL EDTA tubes and processed within 1 h after collection. After double centrifugation (800g for 10 min centrifugation, followed by separation and a second 1600g for 10 min centrifugation), plasma was separated, stored in aliquots and frozen at –80°C until processing. DNA was extracted from plasma and fresh frozen tissue sections using the QIAamp DNA Blood midi kit and the Gentra Purgene Kit (Qiagen, Hilden, Germany), respectively.

### cfDNA concentration and Integrity index

cfDNA fragmentation was assessed by calculating the DNA integrity index as previously described [2]. In brief, it was determined by calculating the ratio of larger (247 bp) versus shorter (115 bp) targets of the consensus sequence of human ALU repeats. The ALU-qPCR result obtained with ALU115 primers was also used to quantify total DNA.

### Methylation specific PCR (MSP)

Purified genomic DNA extracted from tissues and plasma was subjected to bisulfite treatment and DNA purification using the Epitect Bisulfite kit (Qiagen, Hilden, Germany) according to manufacturer’s instructions. A detailed protocol has been previously reported elsewhere [2].

Bisulfite-modified DNA was used as template for Real-Time PCR using a Sybr green-based quantitative MSP. Primers for MSP were designed to specifically amplify either a bisulfite-sensitive, unmethylated strand or a bisulfite-resistant, methylated strand on the *SEPT9* gene promoter region. The web-based software MethPrimer (http://itsa.ucsf.edu/urolab/MethPrimer) was used to select a specific CpG island, which was recently found as the most vulnerable to methylation changes in the adenoma-carcinoma sequence [9].

The sequences of the primer sets were as follows:

M-Fo: TTATTATGTCGGATTTCGCGGTTAAC

M-Rev: AAAATCCTCTCCAACACGTCCG

U- Fo: TAGTTATTATGTTGGATTTTGTGGTTAATG

U- Re: CAAAATCCTCTCCAACACATCCAC (M: methylated, U: unmethylated).

The CpGenome Universal Methylated DNA (Chemicon, Millipore Billerica, MA, USA) was used as 100% methylated (positive) control, whereas DNA extracted from peripheral blood mononuclear cells of normal individuals was used as unmethylated (negative) control.

The PCR reaction mixture was prepared in a final volume of 20 μl, consisting of the following concentrations: 0.375 μM of forward and reverse primers, 250 μM of each dNTP (GE Healthcare, Little Chalfont, UK), 1× HotStart Buffer (Qiagen), 2.5 mM MgCl2, 1.5 units HotStart polymerase (Qiagen), 2 μM SYTO 9 (Invitrogen, Life Technologies, Carlsbad, CA), and 1×ROX reference dye (Invitrogen), 3 μl of bisulfite-modified DNA.

The PCR amplification was performed with precycling heat activation of DNA polymerase at 95°C for 10 min, followed by 40 cycles of denaturation at 95°C for 30 sec, annealing at 64°C for 30 sec and extension at 72°C for 30 sec. An ABI Prism 7500 Sequence Detection System (Applied Biosystems—Foster City, CA, USA) was used.

The PCR product was run on 2% agarose gel to confirm product size and specificity of PCR, and then visualized under UV light. A band of 110 bp was considered as diagnostic of methylation status, whereas a band of 114 bp was considered as diagnostic of unmethylation status.

### KRAS mutation analysis

DNA extracted from tissue and plasma samples was subjected to an allele refractory mutation system qPCR (ARMS-qPCR) for detection of six of the most common mutations in codons 12 and 13 of the *KRAS* gene (G12A, G12D, G12V, G12S, G12C, and G13A). DNA was amplified in a 25 μl reaction mixture containing 0.25 μM of each amplification primer, 200 μM of each dNTP (GE Healthcare, Little Chalfont, UK), 1× HotStart Buffer (Qiagen, Hilden, Germany), 2 mM MgCl_2_, 2 units HotStart polymerase (Qiagen, Hilden, Germany), 2 μM SYTO 9 (Invitrogen, Life Technologies, Carlsbad, CA), 1×ROX reference dye (Invitrogen) and 25 ng DNA. The primer sequences have been previously described elsewhere [10], with the exception of the common reverse primer which has been re-designed in order to shorten the amplicons of both codon 12 (90 bp) and codon 13 (85 bp) (originally of 149 and 144 bp in length). The resulting sequence was as follows: TGTTGGATCATATTCGTCCACA.

The PCR amplification was performed with precycling heat activation of DNA polymerase at 95°C for 10 min, followed by 40 cycles of denaturation at 95°C for 30 sec, annealing at 64°C for 30 sec and extension at 72°C for 30 sec, in a ABI Prism 7500 Sequence Detection System (Applied Biosystems—Foster City, CA, USA). The PCR product of mutated samples was run on 2% agarose gel to confirm the presence of the specific bands.

### Quantitative analysis and analytical performance

Threshold cycles (Ct) were used to calculate methylation rate and mutation load in each sample, according to the following formula: % = 100 / [1 + 2{Ct_met/mut—_Ct_unmet/WT_}] [2,11]. Ctmet and Ctunmet denote threshold cycles specific for the methylated and unmethylated states, whereas Mut and WT refer to mutated and wild-type alleles, respectively. The proportions (%) of methylation rate or mutation load detected in plasma compared to those detected in tissues were expressed as plasma/tissue ratio (p/t ratio).

The median of at least two replicate measurements was calculated for each sample, and then used for statistical analysis. Predefined quality criteria were set, such that measurements with Ct values greater than 38 cycles were excluded.

Since it has been observed that the sensitivity of cfDNA assays can be increased by shortening the size of amplicons [5,6], primers for both analyses were designed to allow the amplification of products smaller than 120 bp. The intra-assay imprecision for the methylation test was 9%. The lower limit of detection of methylated DNA for the MSP assays (assessed using serial dilutions of the Universal Methylated DNA) was 1.5%.

The intra-assay imprecision for the *KRAS* analyses ranged between 2% and 8%, depending on the type of mutation. Cell line DNA admixtures containing the mutation of interest in a normal DNA background were used to evaluate the limit of detection and amplified in the same instrument runs to act as positive controls. The analytical sensitivity of ARMS-qPCR was below 2%, as previously reported [12].

### Statistical analysis

Normality distribution was checked with the Shapiro-Wilk test and continuous variables were reported as median (range) or mean±SD, when appropriate. Statistical analyses and plotting of data were performed using GraphPad Prism (GraphPad Software Inc., San Diego, CA). The diagnostic performance of cfDNA analysis was compared with tumor-tissue analysis (the current gold standard) for its sensitivity and specificity in distinguishing between mutated/hypermethylated and nonmutated/non methylated individuals. The predictive positive and negative predictive values were also calculated with Fisher’s exact test. The rate of concordance between tissue and plasma profiles was determined with agreement test (and values presented as weighted kappa (k) ± standard error). Differences between continuous variables were analyzed by using the Mann-Whitney U test. Correlations were tested with the Spearman correlation. Values of p<0.05 were considered statistically significant.

## Results

Fifty six of 85 patients initially evaluated for their potential inclusion in the study were men, the remaining women (mean age 69±14 years). The tumor stage distribution was as follows: 15 patients were at stage I (17.6%), 35 at stage II (41%), 24 at stage III (28.2%) and the remaining 11 at stage IV (12.9%). Twenty nine out of 85 tumor tissue samples (34%) were positive for one of the six *KRAS* mutations that we have tested. Of these, 22 tumor tissues showed matched mutations in plasma samples. Overall, cfDNA analysis showed 89.4% (76/85) concordance for *KRAS* detection with tumor-tissue analysis (k = 0.753±0.077, p<0.0001). There were nine discordant results among the 85 samples examined. Five results showed a WT genotype for *KRAS*-tested mutations by cfDNA analysis, whereas tumor-tissue analysis showed a *KRAS* G13D mutation (n = 2), a *KRAS* G12D mutation (n = 2) or a *KRAS* G12V mutation (n = 1). Two patients (both at stage II) displayed a *KRAS* G12S and a G12A mutation by plasma analysis, but were determined as WT by tumor-tissue testing. Finally, two patients (both with advanced metastatic CRC) exhibited unmatched mutations between tissue and plasma.

The *SEPT9* promoter methylation was present in 82.3% (70/85) of primary tumor tissue samples. The analysis exhibited 86% (73/85) concordance with cfDNA analysis (k = 0.630±0.092, p<0.0001). Discordant results only concerned patients with aberrant methylation of *SEPT9* in tissue samples and unmethylated plasma samples (n = 12).

The distribution of positive and negative samples in tissue and plasma is shown in Table 1, along with the analytical performance of cfDNA analyses.

**Table 1:** Concordance between tumor-tissue analysis and cfDNA analysis (n = 85).

		Tumour-tissue analysis						
**cfDNA analysis**	**KRAS**	Mutant	WT	Total	SE	SP	PPV	NPV
	Mutant	22	4*	27	85%	93%	85%	93%
	WT	5	54	58				
	Total	27	58	85				
**cfDNA analysis**	**SEPT9**	Methylated	Unmmethylated	Total	SE	SP	PPV	NPV
	Methylated	58	0	58	83%	100%	94%	56%
	Unmmethylated	12	15	27				
	Total	70	15	85				

*: two out of these four patients presented unmatched mutations in tissue and plasma: they showed KRAS G12V mutations by plasma analysis and were determined either G12D or G13D by tumor tissue analysis.

cfDNA: cell-free DNA; WT: wild type; SE: sensitivity; SP: specificity; PPV: positive predictive value; NPV: negative predictive value.

After exclusion of two patients with different *KRAS* genotype in tissue and plasma, the 27 patients (81.5% male) displaying both genetic and epigenetic alterations in tissue specimens (31.8%, 27/85) were considered for further quantitative analyses. In these patients the rate of concordance between tissue and plasma was 93% (25/27) for the epigenetic alteration and 81% (22/27) for the *KRAS* mutation analysis (i.e., two cfDNA samples were negative for the methylation of *SEPT9* and five were negative for the presence of *KRAS* mutations). Among the different KRAS mutations that we have tested, the G12V substitution was the most represented (n = 11), followed by G12D (n = 7) and G13D (n = 7). Finally, one sample exhibited the G12A mutation, whereas the G12S was found in another. Overall, 74% and 26% of mutation sites were located in codons 12 and 13, respectively.

The median *SEPT9* methylation rates in tumour tissues and plasma samples were 64.5% (12.2–99.9%) and 14.5% (0–45.5%), respectively. The median *KRAS* mutation load was 33.6% (1.8–86.3%) in tissues and 2.9% (0–17.3%) in plasma samples. Quantitative data for both genetic and epigenetic alterations according to different clinical pathological characteristics is summarized in Table 2. No significant associations were found with gender, primary tumor site and differentiation status in both tumour tissues and plasma samples. In terms of pathological stage classification, the median methylation rate of *SEPT9* was significantly higher in advanced-stage cancer tissues than in the early stage tissues. A statistically significant correlation was found in between tissue and plasma *SEPT9* methylation rate (r = 0.407, p = 0.035), whereas no association was found between tissue and plasma *KRAS* mutation load (r = 0.092, p = 0.651).

**Table 2:** Associations between SEPT9 methylation rate and KRAS mutation load in tissue and plasma samples according to clinicopathological parameters of CRC patients (n = 27).

		KRAS mutation load (median, range)				SEPT9 mutation load (median, range)			
		tissue	p	plasma	p	tissue	p	plasma	p
**Gender** (mean age±sd)	N (%)		0.2805		0.2099		0.4311		0.9535
Female (71±9)	6 (22.2)	46.8 (14.4–63.7)		8.1 (1.1–13.9)		67.1 (63.7–76.1)		17.8 (0–35.8)	
Male (70±17)	21 (77.8)	27.6 (1.8–86.3)		2.0 (0–17.3)		61.6 (0–35.8)		14.5 (0–45.5)	
**Tumor location**			0.6782		0.4879		0.5613		1.00
Proximal	20 (72)	33.7 (7.7–79.6)		4.0 (0–17.3)		67.1 (12.2–98.1)		13.7 (0–45.5)	
Distal	7 (28)	32.4 (1.8–86.3)		2.3 (0–14.3)		62.6 (18.6–99.9)		23.5 (3.0–35.8)	
**Tumour differentiation**			0.8514		0.1174		0.0656		0.4727
G1/G2	22 (80)	33.0 (1.8–86.3)		1.9 (0–17.3)		62.1 (12.2–99.9)		14.7 (0–45.5)	
G3	5 (20)	33.8 (15.2–62.7)		4.6 (2.3–14.0)		80.8 (64.5–92.7)		10.8 (0–30.0)	
**Pathological stage**			0.1204		0.6612		**0.0009**		0.7340
I/II	14 (51.9)	26.9 (1.8–63.7)		1.9 (0–17.3)		57.2 (15.2–76.0)		15.8 (3.0–40.3)	
III/IV	13 (48.1)	34.7 (11.2–86.3)		4 (0–14.0)		80.8 (12.2–99.9)		12.9 (0–45.5)	

**Fig 1:**
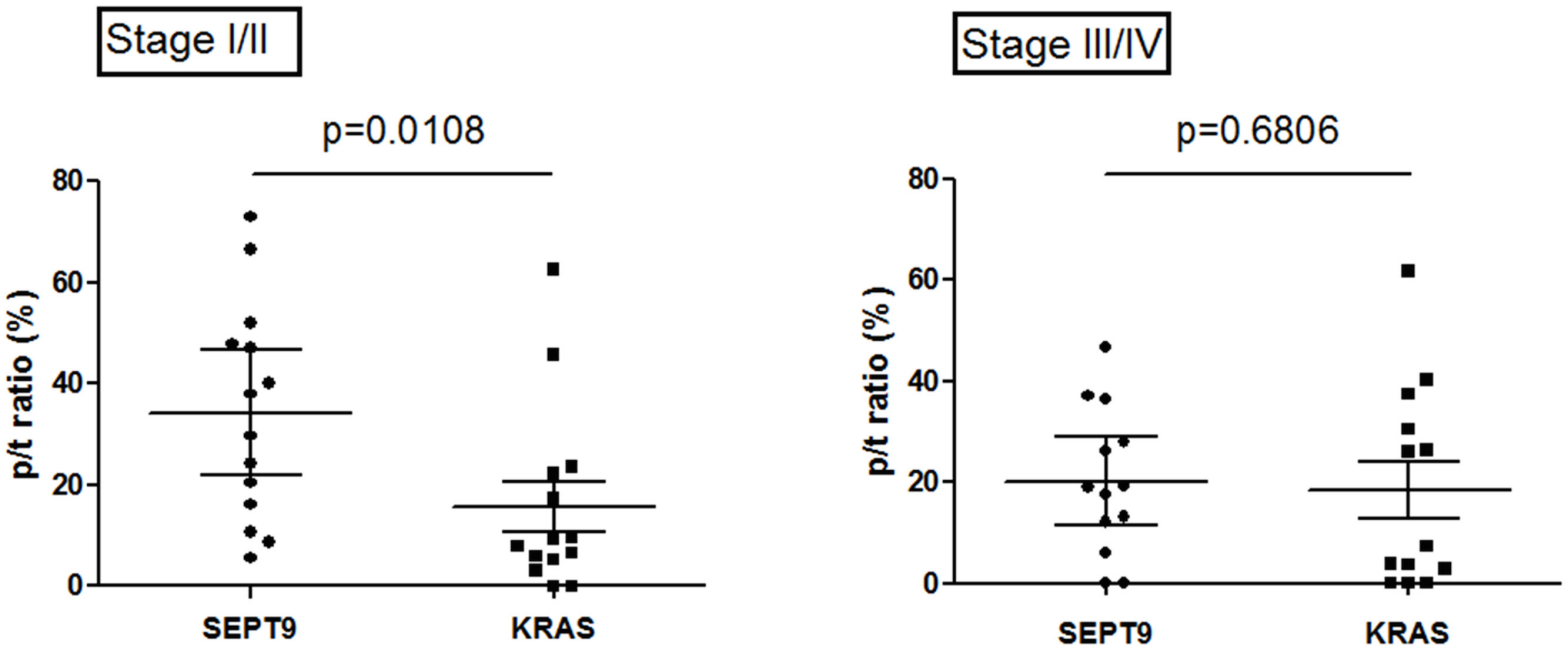
Differences between plasma/tissue methylation rate and mutation load in early and advanced cancer stages.

Additional analyses were performed on p/t ratio of *KRAS* mutation load and *SEPT9* methylation rate, to identify potential differences between genetic and epigenetic degree of transition from tissue to plasma. The p/t ratio of *SEPT9* methylation rate was significantly higher than the p/t ratio of *KRAS* mutation load (24.2% vs 7.9%, p = 0.0228), both parameters showing a wide spectrum of values (range 0–72.9% for *SEPT9* p/t ratio and 0–62.6% for *KRAS* p/t ratio). This finding was almost entirely attributable to the large discrepancy between genetic and epigenetic p/t ratios detectable in early stage cancers (p = 0.0108), since the difference in advanced stage cancers was no longer significant (p = 0.6806) (Fig 1). The concentration of cfDNA in early stages CRC patients (median 30.6 ng/mL, 4.6–66.8) was lower than that in advanced stage patients (80.2 ng/mL, 31.0–195.0; p = 0.0001). The cfDNA was also found to be more fragmented (integrity index: 0.36, 0.0.7–0.85 vs 0.63, 0.33–0.95; p = 0.0163). No significant associations were found between cfDNA parameters and genetic or epigenetic alterations, except for a weak correlation between cfDNA integrity index and *KRAS* mutation load in advanced cancers (r = 0.572, p = 0.040).

## Discussion

Although the use of cfDNA as potential surrogate of cancer genome has been originally suggested more than 30 years ago [13], and the role of liquid biopsy has been evaluated for its predictive and prognostic value in a number of settings with promising results, cfDNA-based cancer tests have not been developed for clinical use so far.

The high degree of fragmentation coupled with the low blood concentration make cfDNA a challenging analyte under a technical perspective. Moreover, the still uncertain kinetics of tumor-related cfDNA release into the bloodstream and the genetic composition changes during progression both contribute to make cfDNA a”hard to read”analyte, even under a biological perspective.

The results of our study, other than confirming that liquid biopsy predicts alterations of tumor tissues, are consistent with the hypothesis that some differences may exist among the rate at which genetic and epigenetic alterations move from tissue to plasma.

In order to make results less vulnerable to technical interference and make genetic and epigenetic data reliable and directly comparable, we adopted a number of methodological expedients adapted from recent publications. First, the analysis was performed in plasma since this biological matrix represents a better source of cfDNA than serum [1, 6]. Then, we used relative short amplicons for both determinations, and this was due to the fact that the length of the fragment may influence the sensitivity of detecting mutation and methylation[5, 14, 15]. We have also assured a high level of sensitivity of the epigenetic assay by targeting a specific CpG island, which has been recently found to display the highest susceptibility to methylation changes in the adenoma-carcinoma sequence [9]. Finally, according to the American Society for Clinical Oncology and National Comprehensive Cancer Network (NCCN), a high level of detection rate has been obtained for *KRAS* mutation analysis by targeting hotspots in codon 12 and 13, which are known to account for approximately 95% of all mutations [16].

In the present study, a methylation specific qPCR and an ARMS-qPCR based methods were used for *SEPT9* methylation and *KRAS* mutation analyses, respectively. Due to important technological advances, new methods such as digital PCR [17], Inteplex qPCR [14] BEAMing technology [18], MethyLight quantitative or MethyLight digital PCR [19] and new deep sequences approaches [20] are now available, thus allowing absolute quantification of mutant or methylated alleles at very low frequencies and with lower imprecision than those reported here. However, the assays that we used in this study are more widely available in clinical laboratories, and are also characterized by optimal sensitivity, being able to detect at least 2% alteration in a normal background [21]. Even more importantly, the analytical performance of genetic and epigenetic assays were very similar in terms of sensitivity and precision, thus allowing direct comparison of data from different alterations.

The first part of the study, performed on the entire cohort of 85 CRC patients, substantially confirmed previous evidence that analysis of *KRAS* and *SEPT9* in plasma may be seen as a reliable alternative to the tissue. The status of *KRAS* is generally used as predictive marker of response to established epidermal growth factor receptor (EGFR) inhibitors due to the fact that mutant *KRAS* is associated with resistance to anti-EGFR monoclonal antibody immunotherapy with agents such as centuximab or panitumumab [22,23]. Conversely, aberrant methylation in the promoter region of the *SEPT9* gene has been convincingly proposed as sensitive and specific biomarker for early non-invasive diagnosis of CRC [24].

By following the suggestions recently proposed by Wasserkort and coauthors [9], and thus targeting a specific CpG island on the promoter of the *SEPT9* gene, we found a very high number of hypermetylated tissues samples (82%), to a higher extent than previously reported in the literature (usually ranging between 78 and 81%) [25]. The results obtained in matched plasma samples revealed high global concordance (86%) and specificity (100%) compared with tumour-tissue analysis. In the same sample, a *KRAS* mutation was detected in 34% of patients, in line with data obtained in other cohorts of unselected CRC patients [10,26]. The corresponding analysis of plasma samples also revealed a high degree of concordance (89.4%) and specificity (93%) compared with tissue. Most of the studies comparing the results from a cfDNA assay with tumour-tissue analysis reported a much lower diagnostic performance, with values of specificity constantly lower than 80% [27–29]. As an exception, only two recent studies reported values of specificity comprised between 95.3% [30] and 98% [14].

In the second part of the study, we analysed the rate of concordance between tissue and plasma mutation load and methylation rate, and results obtained with the two assays were then compared. In the subgroup of 27 patients harbouring tissue genetic and epigenetic alterations, the *KRAS* mutation load varied from 1.8% to 86.3% (almost 48-fold), thus showing a higher interindividual heterogeneity than the *SEPT9* methylation rate, which varied from 12.2% to 99.9% (i.e., approximately 8-fold). In the transition from tissue to plasma, five samples became WT for the mutation status and two were no longer hypermethylated. The degree of methylation moving from tissue to plasma was almost 3 times higher than the rate of mutation load as resulting from the comparison of the two p/t ratios (24.2% vs 7.9% for p/t ratio of *SEPT9* methylation rate and *KRAS* mutation load, respectively). In agreement with recent reports, this finding might be explained by the intratumoral heterogeneity of the primary tumour, which preferentially impairs genetic rather than epigenetic analysis [7, 31]. Nevertheless, since the discrepancy found between the two p/t ratios is exclusively attributable to data obtained in early stage cancers whereas clonal evolution usually occurs when metastasis is developing, the tumour clonality would only partially explain our findings [32].

For the *KRAS* analysis, comparable values of mutation load were obtained between early and advanced cancers in both tissue (26.9% vs 34.7%) and plasma samples (1.9% vs 4%), so that the p/t analysis did not reveal significant difference according to tumour stages (8.6% vs 7.3%). Conversely, a statistical significant difference was found for the *SEPT9* methylation analysis between p/t ratio in early and advanced cancers (33.8% vs 19.0%, p = 0.0108). This variance was entirely attributable to a discrepancy in the methylation rate detected in tissues (57.2% vs 80.8%, p = 0.0009), since no differences were found in plasma samples (15.8% vs 12.9% for early vs advanced stages). Thus, the transition of DNA harbouring the epigenetic alteration into the circulation in early stage cancers is seemingly more consistent than the transition of DNA harbouring a *KRAS* mutation. According with the most recent literature data, this evidence could be interpreted as resulting from differences in tissue types involvement previously observed for CRC genetic and epigenetic signatures [33]. In particular, while the *SEPT9* aberrant methylation originates in epithelial cells and is then rapidly transferred to stromal cells [9], the *KRAS* mutations harboured by epithelial compartment are not shared by stromal cells [34]. Accordingly, the molecular cross-talk between tumour epithelium and stroma occurring for the *SEPT9* epigenetic alteration might facilitate the transition of aberrant DNA from primary tumour to the circulation.

In addition, the overall lower degree of mutation load with respect to the methylation rate detected in CRC tissues (26.9% vs 57,2% in early stage cancers) might have contributed to enhance the dilution effect of wild type-*KRAS* DNA in the circulation.

In conclusion, the results of the present study confirm that cfDNA analysis represents a suitable strategy for comprehensive analysis of tumor genetic and epigenetic profiles, even using routine methods. Most importantly, we provided first evidence that the rate to which tumour derived cfDNA can be detected into the circulation not only depends on the sensitivity of methods used and complexity of release kinetics, but also on the nature of the single alteration. In an era characterized by increasing use of comprehensive gene expression studies of solid tumours to elucidate the complexity of tumour tissues and heterogeneity of cell phenotypes, our study emphasizes the need to better characterize cancer-specific genetic and epigenetic signatures according to different tumour compartments, so that the significance and clinical value of cfDNA assessment can be ultimately improved.

Additional confirmatory studies may support the hypothesis already suggested by some authors, according to which analysis of cfDNA represents a valuable alternative to tissue analysis but may also become the first choice for both genetic and epigenetic tumour characterization by providing a better overall portrait of malignant diseases [5, 14, 29, 30].
